# Preparation, Characterization, and Evaluation of Macrocrystalline and Nanocrystalline Cellulose as Potential Corrosion Inhibitors for SS316 Alloy during Acid Pickling Process: Experimental and Computational Methods

**DOI:** 10.3390/polym13142275

**Published:** 2021-07-12

**Authors:** Arafat Toghan, Mohamed Gouda, Kamal Shalabi, Hany M. Abd El-Lateef

**Affiliations:** 1 Chemistry Department, College of Science, Imam Mohammad Ibn Saud Islamic University (IMSIU), Riyadh 11623, Saudi Arabia; arafat.toghan@yahoo.com; 2 Chemistry Department, Faculty of Science, South Valley University, Qena 83523, Egypt; 3 Department of Chemistry, College of Science, King Faisal University, Al Hofuf, Al-Ahsa 31982, Saudi Arabia; 4 Chemistry Department, Faculty of Science, Mansoura University, Mansoura 35516, Egypt; dr-kamal@mans.edu.eg; 5 Chemistry Department, Faculty of Science, Sohag University, Sohag 82524, Egypt

**Keywords:** natural polymers, nanocrystalline cellulose, corrosion protection, DFT calculations, green synthesis

## Abstract

Converting low-cost bio-plant residuals into high-value reusable nanomaterials such as microcrystalline cellulose is an important technological and environmental challenge. In this report, nanocrystalline cellulose (NCC) was prepared by acid hydrolysis of macrocrystalline cellulose (CEL). The newly synthesized nanomaterials were fully characterized using spectroscopic and microscopic techniques including FE-SEM, FT-IR, TEM, Raman spectroscopy, and BET surface area. Morphological portrayal showed the rod-shaped structure for NCC with an average diameter of 10–25 nm in thickness as well as length 100–200 nm. The BET surface area of pure CEL and NCC was found to be 10.41 and 27 m^2^/g, respectively. The comparative protection capacity of natural polymers CEL and NCC towards improving the SS316 alloy corrosion resistance has been assessed during the acid pickling process by electrochemical (OCP, PDP, and EIS), and weight loss (WL) measurements. The outcomes attained from the various empirical methods were matched and exhibited that the protective efficacy of these polymers augmented with the upsurge in dose in this order CEL (93.1%) < NCC (96.3%). The examined polymers display mixed-corrosion inhibition type features by hindering the active centers on the metal interface, and their adsorption followed the Langmuir isotherm model. Surface morphology analyses by SEM reinforced the adsorption of polymers on the metal substrate. The Density Functional Theory (DFT) parameters were intended and exhibited the anti-corrosive characteristics of CEL and NCC polymers. A Monte Carlo (MC) simulation study revealed that CEL and NCC polymers are resolutely adsorbed on the SS316 alloy surface and forming a powerful adsorbed protective layer.

## 1. Introduction

SS316 steel alloy has widespread usage in chemical, automotive, petroleum, and oil production [1]. Its mechanical, physical, and structural characteristics make it appropriate for use as a suitable component, or part the SS316 steel alloy parts might corrode in acidic, alkaline, or neutral mediums [2]. SS316 steel alloy is vulnerable to harsh circumstances such as acidic or saline solutions [3]. Numerous approaches have been applied for the protection of these alloys from serious corrosion issues according to the economic aspects and service type [4,5,6]. The corrosion is an electrochemical process in which anodic and cathodic half-processes occur with matching kinetics. The cathodic half-reaction can be different phenomena such as hydrogen evolution, and the anodic half-reaction is metal dissolution. Obstructing both reactions can reduce the complete corrosion development [7].

Polymeric materials are one of the favorable applicants for corrosion protection due to their precise features. These materials cover all the plain parts of the electrode interface and defend it from the aggressive solution. Several polymeric materials have been applied as inhibitors for steel alloy corrosion including polyacrylamide derivatives [8], polyacrylic acid derivatives [9], polyethyleneimine derivatives [10], polyvinylsilsesquioxanes [11], and polyvinylpyrrolidone (PVP) [12]. 

Microcrystalline cellulose, which comprises linear chains of β-D-glucose units connected by β-1,4-glycosidic linkages, is a plentifully accessible polymeric biomaterial in nature [13,14,15,16]. The source sequence of MCC is also almost infinite; it tends to be artificially extracted from the plant via treatment with an alkaline medium followed by bleaching actions [16,17]. Cellulose nanocrystals can be prepared by acid hydrolysis of microcrystalline, which yields cellulose nanocrystals [18,19,20]. Recently, nanocellulose has acquired the insuperable interest of analysts throughout the planet because of its intriguing and novel properties.

For instance, microcrystalline can be an incredible structure block for material functionalization or as support filler because of its biodegradability, biocompatibility, high mechanical strength, and enormous surface region. This opens up plenty of opportunities for its application in different fields, for example, food bundling, auto, nano-remediation, and different ventures [21,22,23,24]. Although nanocellulose can be obtained from an assortment of plant sources, most examinations directed at nanocellulose are from commercial microcrystalline cellulose removed from cotton and wood mash [25,26]. Extra data on the crucial parts of nanocellulose, for example, on its construction and properties when it is extricated from various sources, are unquestionably significant. 

**Figure 1:**
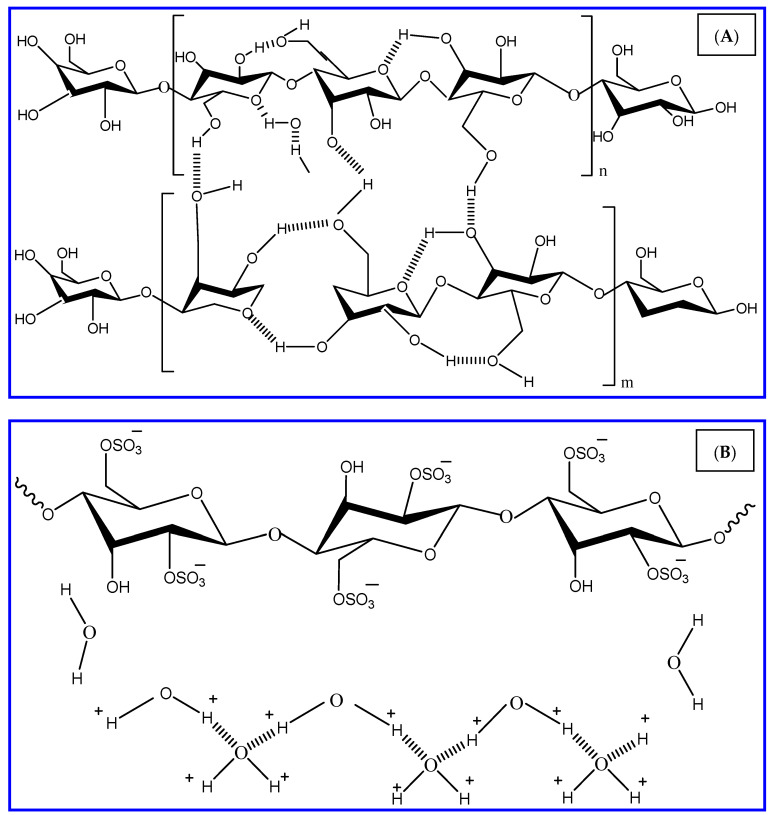
The chemical structure of the used inhibitors (**A**) CEL and (**B**) NCC.

The examinations led in this paper were pointed towards assessing the distinctions in properties of microcrystalline cellulose (CEL) and nanocrystalline cellulose (NCC) (Figure 1). The characteristics of both CEL and NCC were described by utilizing field emission scanning electron microscope (FESEM), Fourier transform infrared (FTIR) spectroscopy, Raman spectroscopy, transmission electron microscopy, and surface area analysis. The comparative inhibition performance of CEL and NCC on the SS316 alloy corrosion during the acid pickling process was studied by weight loss and electrochemical (PDP and EIS) measurements. The SEM technique was used to investigate the surface characterization of the SS316 alloy in the inhibited and blank systems. DFT-based calculations and MD simulations were accomplished to associate the quantum chemical parameters of the used CEL and NCC and their experimental protection capacity.

## 2. Experimental Part

### 2.1. Materials

Cellulose powder with molecular weight 10,000 was purchased from Fluka AG (Buchs, Switzerland) laboratories, sulfuric acid (95–97%), and 2-propanol (99.8%) from Sigma-Aldrich (St. Louis, MI, USA).

### 2.2. Synthesis of Nanocrystalline Cellulose (NCC)

Nanocrystalline cellulose (NCC) was prepared by acid hydrolysis of 10.0 g commercial microcrystalline cellulose (CEL) with a 65 wt% sulfuric acid at 50 °C for 1 h in a water bath with mixing. Following hydrolysis, the prepared suspension was diluted with distilled water 10 times to end the reaction. The suspension was then washed over and again with water and centrifugation (6000 rpm, 10 min for each cycle) until the pH of the supernatant was higher than 1. The samples were consequently dialyzed alongside water for three days. At last, the samples were dried through vacuum freeze to acquire NCC powders.

### 2.3. Characterization

The readied CEL and NCC were described by FT-IR spectroscopy utilizing Fourier transform infrared instrument (Agilent Innovations, cary 630, Santa Clara, CA, USA). The spectra were dissected inside the reach 4000–400 cm^−1^. Surface morphology was concentrated by a field emission scanning electron microscope (FESEM JXA-840 and electron test microanalyzer JOEL). The transmission electron microscope (TEM) was utilized to research the dispersion and the average diameter measurement of the prepared samples (TEM-ZEISS-EM-10-GERMANY). Raman spectroscopy was utilized to affirm the synthetic virtue of the readied nanocomposite, utilizing a Raman spectrometer (HORIBA SCIENTIFIC, SUA). The Brunauer–Emmitt–Teller (BET) strategy for a completely programmed explicit surface area and pore size analyzer (Ji Nan RunZhi Innovation, China) was utilized to quantify the particular surface area of the readied samples.

### 2.4. Preparation of Electrode Coupons and the Inhibitor Solutions 

The chemical compositions (wt.%) of the cylinder stainless steel 316 electrode used for this investigation are 0.07% C, 0.55% Mn, 0.03% Si, 0.02% P, 0.06% S, 1.69% Cr, 0.09% Mo, 1.04% Ni, 0.01% Ti, and the rest Fe. Before the electrochemical tests, the stainless steel electrode was embedded in epoxy resin, leaving a 1.0 cm^2^ surface area exposed to the corrosive medium. Before usage, the electrode specimens were mechanically ground with a sequence of SiC emery papers of different grits (400, 600, 800, 1000, and 1500). These were then degreased with acetone and rinsed with bidistilled water before immersion in the solution.

A stock solution of analytical grade hydrochloric acid (37%) was utilized to prepare an aqueous solution of HCl (2.0 M HCl) by the dilution method. After that, CEL and NCC solutions (25–200 mg/L) were separately prepared in the acid solution and applied as inhibitors for this investigation.

### 2.5. Electrochemical and Weight Loss Measurements 

The electrochemical assessment of the electrode sample accomplished by a 3-electrodes arrangement is described here. The electrodes are a Pt-sheet as a counter electrode, a silver/silver chloride (Ag/AgCl) as a reference electrode, and an SS316 alloy as a working electrode. The electrode arrangement connected to the Gamry Galvanostat/Potentiostat/ZRA electrochemical workstation, based on ASTM standard method [27], was utilized. The corrosion inhibition features of the SS316 alloy electrodes, in the acidic medium (2.0 M HCl), were investigated by the electrochemical methods: potential-time, EIS, and PDP, respectively. Prior to every run, the SS316 alloy was immersed in the corrosive medium for 40 min until a stable state is reached at open circuit potential (*E*_OCP_). EIS experiments were completed at the EOCP by assessing the frequency range of 100 kHz to 0.1 Hz with a signal amplitude of 10 mV. A scan rate of 0.25 mV s^−1^ and a voltage ranging from −0.25 to +0.25 V from *E*_OCP_ were used for the PDP. Weight loss investigation was completed at different temperatures based on the standard process: American Society for Testing and Materials (ASTM) G1–03 technique [27]. All corrosion tests were reiterated three times to confirm the accuracy of the experimental findings. The temperatures of corrosion tests were extended from 25 to 55 °C.

### 2.6. Surface Characterization

The SS316 alloy surfaces in the presence and absence of the titled inhibitors were inspected by the SEM device (JSM-6610 LV model) at 20 kV (operating voltage) and 10 mA (irradiation current).

### 2.7. DFT Calculations and MC Simulations

Computational studies were completed via Accelrys Materials Studio 7.0 using the DMol^3^ module for DFT calculations and adsorption locator module for MC simulations. The structure of the repeated unit for the microcrystalline cellulose structure (CEL) and nanocrystalline cellulose (NCC) was optimized in DFT calculations by applying generalized gradient approximation (GGA)/Becke-Lee-Yang-Parr (BLYP) functional with double numerical basis set plus polarization function (DNP) basis set and COSMO solvation controls [28]. For MC simulations, the most appropriate adsorption arrangements of the CEL and NCC molecules on the Fe (1 1 0) surface are revealed by operating the adsorption locator module based on Monte Carlo searches to assess the protective efficacy of inhibitor molecules [29]. The adsorption of inhibitor molecules, water molecules, and the surface of Fe (1 1 0) was achieved in a simulation box (32.27 Å × 32.27 Å × 50.18 Å) with the assigned COMPASS force field [30]. Furthermore, all the inputs, outputs, and calculations of computational studies were described in our formerly published studies [28,29]. 

## 3. Results and Discussions

### 3.1. Spectroscopic and Microscopic Analysis 

#### 3.1.1. FT-IR

**Figure 2:**
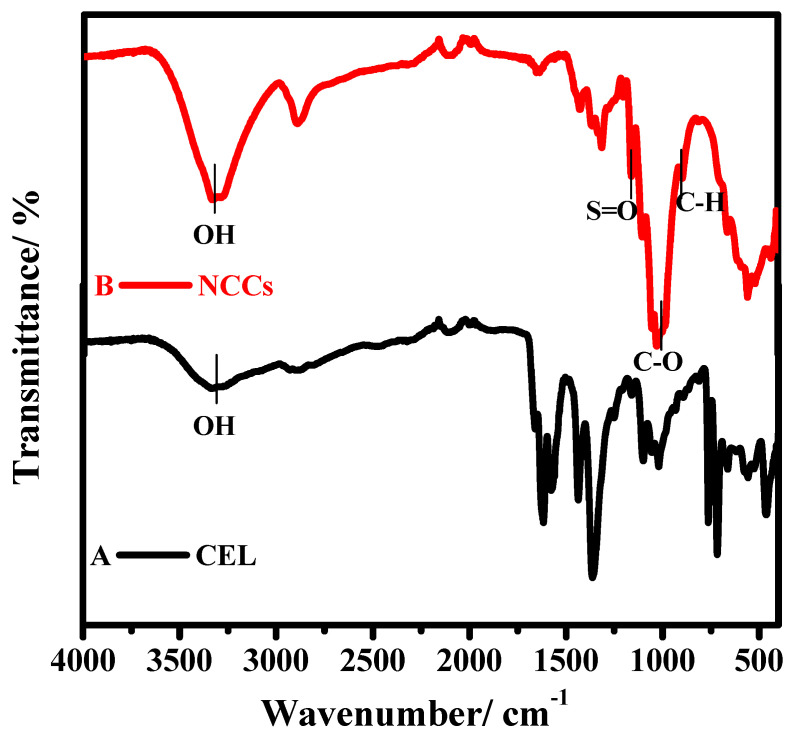
FTIR spectra of (**A**) CEL and (**B**) NCC.

FT-IR spectroscopy was utilized to acquire an understanding of the progressions in the compound constructions and the properties of a material. As portrayed in Figure 2, the −OH group that appeared in the range of 3700 to 3100 cm^−1^ of NCC was more extreme and smaller when contrasted with CEL. This marvel depicts the improvement in the strength of hydrogen bonds of the NCC that came about because of the disposal of the undefined constituents that thus brought about an increment in crystallinity, and this discovery will be upheld in the later segment [31]. Pinnacles occurring at 1430, 1321, 1062, and 897 cm^−1^ are typical peaks of cellulose [32]. In contrast with CEL, NCC occurs in a higher cellulosic content than CEL because of the expansion in band intensity at 1062 cm^−1^ (C–O extending) and 897 cm^−1^ (C–H rock vibration) [32]. The band at 1205 cm^−1^, which is ordinarily present in NCC because of the arrangement of S=O linkage from the esterification interaction during hydrolysis via sulfuric acid, was available [33].

#### 3.1.2. Raman Analysis

**Figure 3:**
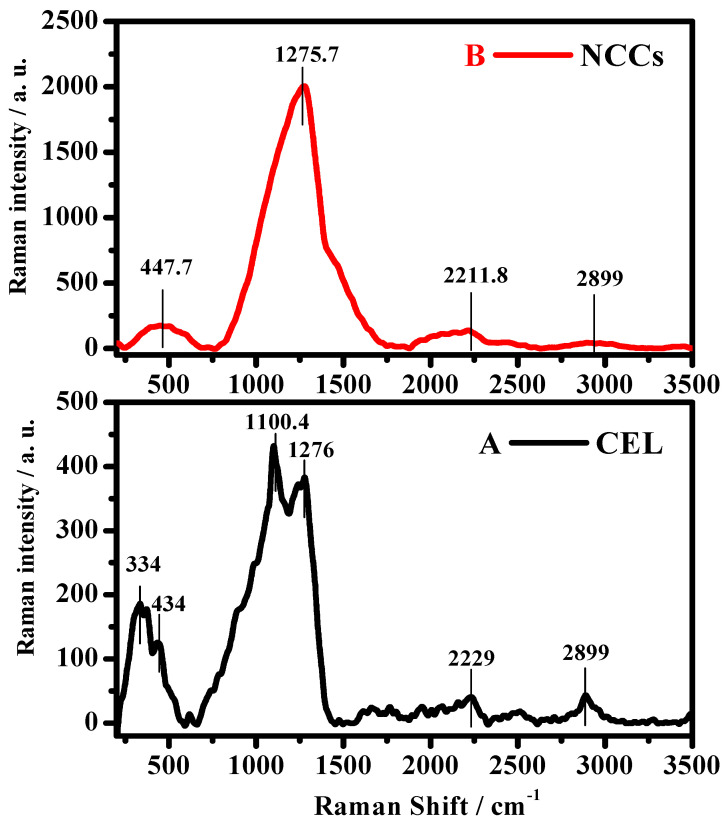
Raman spectra of (**A**) CEL and (**B**) NCC.

The bands under 1700 cm^−1^ in Raman spectra represent the backbone of cellulose. Resilient and thoughtful bands are for the most part noted in the scope of 100 to 1700 cm^−1^. The area over 2700 cm^−1^ is mostly more delicate to hydrogen bonding [34]. A complete variety of the Raman spectra (50–4000 cm^−1^) for two samples, CEL and NCC, appeared in Figure 3A,B, respectively. It very well may be seen that there is a band at 1700 cm^−1^. The Raman range for CEL (Figure 3A) shows peaks situated at ~334, ~434, ~1100, 1276, 2229, and 2889 cm^−1^. The acid hydrolyzed NCC shows diapering tops situated at ~334 and ~1110 cm^−1^ (Figure 3B). A peak situated at ~2889 cm^−1^ can be credited to C-H/CH_2_ extending vibrations. No top between 3200 and 3500 cm^−1^, which would compare to OH extending vibrations, is available in any of the CEL and NCC samples. This would infer that every one of the samples was appropriately dried and kept in an excellent dry condition. It very well might be sensible to investigate the subtleties of the Raman spectra (Figure 3A,B) recorded in the otherworldly scope of 100–1700 cm^−1^. The pinnacle forces for the CEL are seen in Figure 3A and the acid-treated samples in Figure 3B. The peak at ~1276 cm^−1^ addresses the HO-C bending mode. The band at ~447 cm^−1^, addressing the trademark mode for shapeless cellulose, is additionally present in CEL and NCC with shifting power [35] (Figure 3A,B). The skeletal bending methods of CCC, COC, OCC, and OCO are for the most part predominant in the 150–550 cm^−1^ area. Methane twisting (CCH, COH) and the development of CC and CO gatherings inside the glucopyranose ring units may be engaged with such cases. One may notice the presence of the above peaks in this 150–550 cm^−1^ district, the samples with differed power. The most noticeable differentiation in NCC in the 150–1220 cm^−1^ district is the shortfall of bands around 334 and 1110 cm^−1^. This would likely show -COH twisting [36]. The band showing up in the spot of ~1200 cm^−1^ could be attributed to C–C and C–O extending modes and a few measures of HCC and HCO bending styles. The overhead band obtained was unmistakably more grounded for the sample treated with acid (Figure 3B). The expansion in the power of the bands of NCC could be credited to an increment in the crystallinity of cellulose and sizes of crystallite.

#### 3.1.3. FE-SEM and TEM

**Figure 4:**
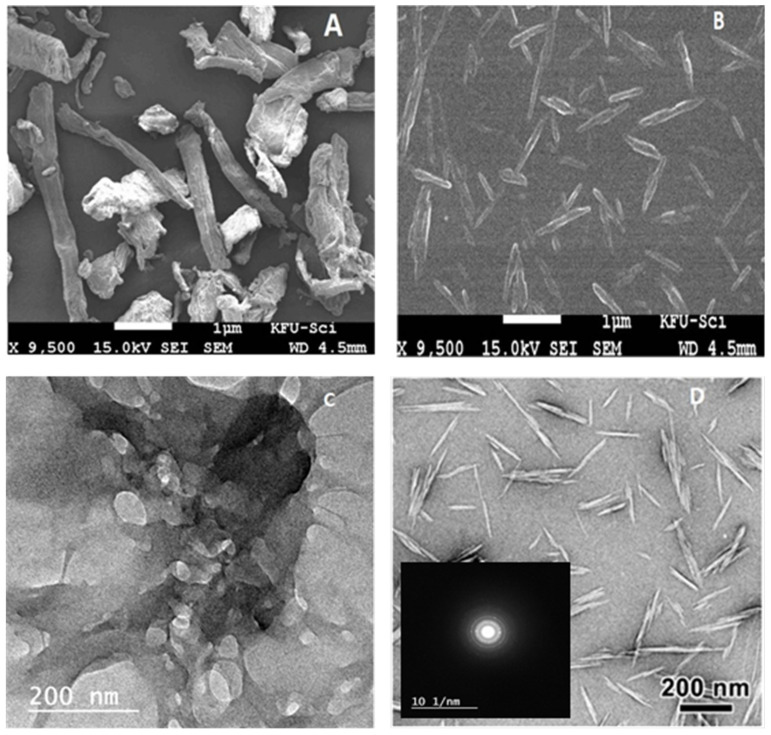
FE-SEM image of (**A**); CEL and (**B**); NCC and TEM image of (**C**); CEL and (**D**); NCC and D inset corresponding SAED pattern of NCC.

Figure 4A shows the gathered constructions of CEL that orchestrated like chips because of solid fascination using hydrogen bonding among the surface hydroxyl gatherings. By contrasting Figure 4A,B with NCC, it tends to be seen that there is a significant decrease in size after acid hydrolysis because of the effective evacuation of the amorphous region [37]. As demonstrated in Figure 4A, the crude CEL shows an amorphous network fibrous structure, while after the acid treatment, the morphology of NCC was effectively obtained because of the evacuation of the undefined district of CEL. This reality is additionally affirmed by TEM. There is a significant decrease in molecule size, showing the surface drawing and disintegration of the hydrolysis interaction. Furthermore, Figure 4C,D show the TEM pictures of CEL and NCC. The nanoscale particles isolated from CEL all present an elongated rod shape. The NCC was approximately 100 to 200 nm long and 10 to 20 nm wide. The components of nanocrystalline cellulose rely emphatically upon the handling strategies and hydrolysis conditions. Mostly, more grounded acidity, longer response time, and higher temperature may yield more limited NCC. Figure 4D shows that the NCC has a rod-like construction alongside round formed particles of 10–15 nm. In addition, selected-area electron diffraction (SAED) shows typical ring patterns, indicating the polycrystalline nature of NCC.

#### 3.1.4. BET Analysis

The adsorption of gases on solids can be utilized to decide the accessible surface area of the solid material. The procedure depends on uncovering the solid material to expand the halfway pressing factors of the examining gas and estimate the measure of gas adsorbed on a superficial level to make an adsorption isotherm. At a specific pressing factor, a monolayer of gas covers every one of the surfaces of the sample. Now, the particular surface region can be resolved utilizing a model created by Brunauer, Emmet, and Teller. The BET surface areas of pure CEL and NCC were determined to be 10.41 and 27 m^2^/g, respectively.

### 3.2. Corrosion Inhibition Data 

#### 3.2.1. Weight Loss Analysis

**Figure 5:**
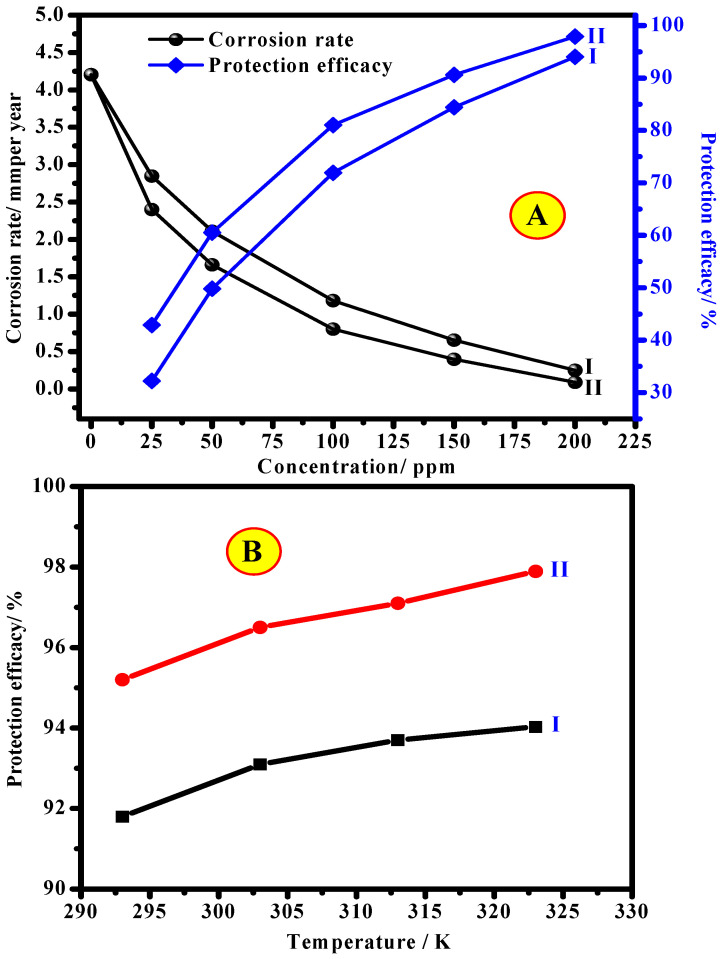
Change of corrosion rate and protection capacity for SS316 alloy with (I) NCC and (II) CEL concentrations at 50 °C (**A**) and effect of temperature on the protection capacity of SS316 alloy in 2.0 M HCl containing 200 ppm of (I) CEL and (II) NCC (**B**).

The weight-loss examination was performed at different temperatures (20–50 °C), and the result is displayed in Figure 5A,B. The inhibited system meaningfully showed lesser values of corrosion rate (*CR*) in comparison to the uninhibited medium, and the CR declines with a rise in CEL and NCC concentration. Concerning the SS316 alloy, the CR drops from 4.203 mm/year in 2.0 M HCl to 0.251 and 0.088 mm/year in 200 mg/L of CEL and NCC, respectively. The observed reduction in the CR is related to the CEL and NCC adsorption onto the substrate surface to process a defensive inhibitor layer on the electrode interface. This adsorbed layer prevents the corrosive ions from reaching the alloy surface. The experiential greater protection efficacy (Figure 5A) with incremental CEL and NCC dose is the significance of the cumulative amount of adsorbed inhibitor on the electrode interface. The maximum dose (200 mg/L) demonstrated the optimum protection capacity of over 94.3% and 97.9% for CEL and NCC, respectively. Additionally, the impact of different solution temperature on the CR and protection act of the CEL and NCC molecules was experienced using a blank (uninhibited medium) and 2.0 M HCl containing 200 ppm of CEL and NCC, at a temperature range of 20–50 °C, respectively. The findings indicated that the CR was increased considerably for the uninhibited medium (Blank HCl) with increasing solution temperature. The minor rise in the protection efficacy with the temperature at the 200 mg/L CEL and NCC (Figure 5B) proposes that the adsorption of CEL and NCC at this dose could include the chemical adsorption route. In the chemical adsorption mechanism, the strength of interaction among CEL and NCC molecules is very robust. much sturdier than the Vander Waals forces of attraction (physical adsorption). Consequently, the protection efficiency considerably rises from lesser inhibitor doses to higher concentrations.

#### 3.2.2. Open Circuit Potential

**Figure 6:**
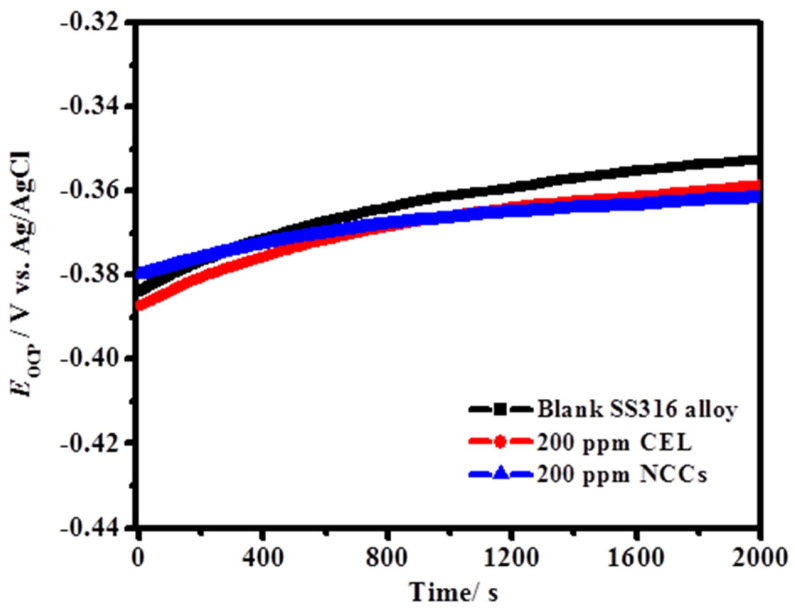
Fruition of the OCP versus time for SS316 alloy in 2.0 M HCl at the maximum-tested dose of (200 ppm) CEL and NCC at 323 K.

The change of the SS316 alloy *E*_OCP_ against the immersed time during 2000 s for the blank medium and the highest-investigated dose (200 mg/L) of the CEL and NCC inhibitors at 323 K is exemplified in Figure 6. It was observed that the insertion of the tested NCC and CEL inhibitors prompts a shift in *E*_OCP_ (i.e., *E*_cor_). According to the diagram displayed in Figure 6, it could be seen that the SS316 alloy specimen can attain a quasi-steady *E*_OCP_ in under~ 1500 s. Consequently, 25 min of *E*_OCP_ extent was expected earlier to perform entirely PDP and EIS measurements in this study.

#### 3.2.3. Tafel Plots 

**Figure 7:**
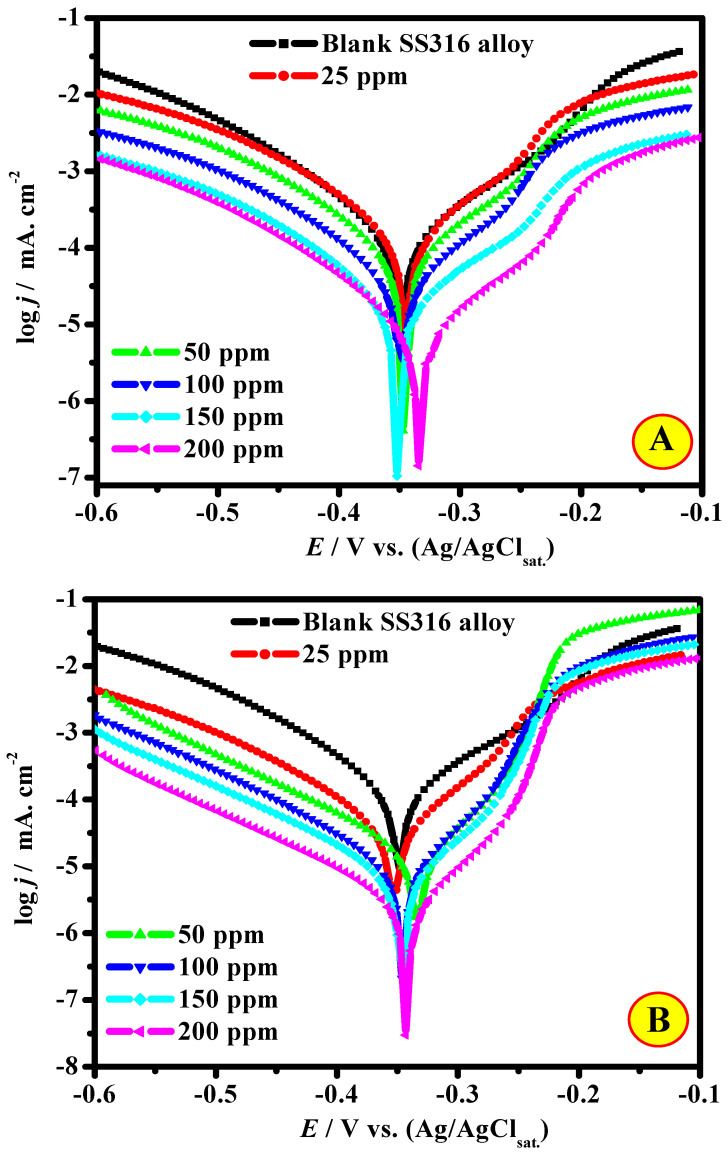
PDP plots of SS316 alloy in 2.0 M HCl solution with and without various concentrations of (**A**) CEL and (**B**) NCC at 50 °C.

The corrosion impedance of the SS316 alloy was investigated by the PDP method in 2.0 M HCl medium and in the occurrence of CEL and NCC inhibitors at 323 K. Figure 7A,B display log(*j*)-*E* profiles for SS316 alloy in blank and inhibitor-containing mediums at a sweep rate of 0.2 mV/s. The CEL and NCC inhibitors impede the SS316 alloy corrosion through the shifting of both the cathodic and anodic branches to lower current values. Some PDP parameters such as corrosion potential (*E*_cor_), corrosion current density (*j*_cor_), anodic and cathodic Tafel slopes (*β*_a_, *β*_c_), and the protection efficacy (*ζ*_PDP_/%) calculated from these plots are summarized in Table 1. The protection efficacy (*ζ*_PDP_/%) and surface converge (*θ*) were intended using *j*_cor_ in the absence ($j_{cor}^{0}$) and presence ($j_{cor}^{i}$) of inhibitors based on the following Equation [38]:

(1) $$\zeta_{\mathrm{PDP}}=\theta \times 100=\left(\frac{j_{cor}^{0}-j_{cor}^{i}}{j_{cor}^{0}}\right)\times 100$$

The inhibition capacity of the investigated inhibitors follows the order NCC (96.3%) > CEL (93.1%) at 200 ppm inhibitors dose. The difference in inhibition capacity of CEL and NCC inhibitors could be clarified according to their dissimilar chemical configurations. It could be observed from this plot that the *β*_a_ and *β*_c_ in the occurrence of NCC and CEL inhibitors slightly change compared to the uninhibited cathodic lines. Correspondingly, all plots increase to parallel appearances, demonstrating that the prepared CEL and NCC compounds do not modify the mechanism of hydrogen evolution [39]. In other words, the investigated compounds can diminish the hydrogen ions by covering the efficient reaction positions at the SS316 alloy interface, developing, consequently, a protecting layer. Furthermore, the values of *β*_c_ did not display a large change with the rise of the inhibitor, which designates that the hydrogen reaction reduction is examined based on the pure mechanism activation [40].

An appearance at Figure 7A,B indicates that the oxygen evolution and/or hydrogen reduction at the cathodic site and the SS316 alloy anodic dissolution were inhibited in the existence of CEL and NCC compounds and *j*_cor_ upsurge with their dose. This phenomenon suggests that CEL and NCC molecules impede the corrosion of the SS316 alloy by adsorbing at the efficient sites on the metal interface, and the surface coverage (*θ*) at metal/electrolyte interface upsurges with increments in the number of inhibitor molecules [41]. The lessening in the SS316 alloy corrosion revealed by CEL and NCC molecules could be characterized as cathodic, anodic, or mixed-type according to the movement in their *E*_cor_ values with relation to the *E*_cor_ value of the uninhibited system (blank). Some recent works show that if the change in the *E*_cor_ value is > 0.085 V, the compounds could be considered mixed-type inhibitors. In circumstances where the movement in *E*_cor_ value is < 0.085 V, inhibitors could be categorized as cathodic or anodic type, depending on the way of movement. From the findings presented in Table 1, the extreme changes in *E*_cor_ were 0.016 V with CEL and 0.018 V with NCC, signifying that both compounds performed as mixed-type inhibitors.

#### 3.2.4. EIS Studies 

**Figure 8:**
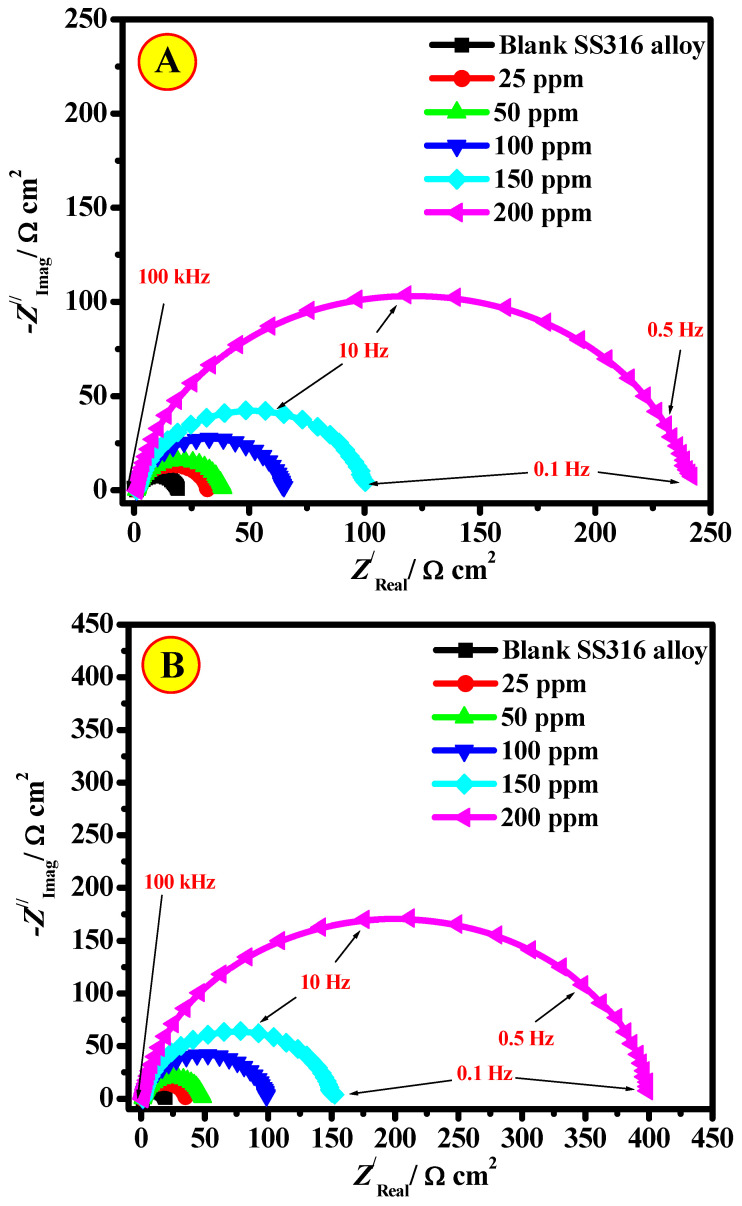
Nyquist diagrams of SS316 alloy in 2.0 M HCl in the absence and the presence of various concentrations of (**A**) CEL and (**B**) NCC, at T = 323 K.

To obtain more evidence about the mechanism of corrosion processes and approve the earlier findings attained from weight loss and PDP measurements, EIS analysis was achieved. Therefore, the Nyquist diagrams of the SS316 alloy specimens in 2.0 M HCl in the existence and lack of the CEL and NCC molecules are presented in Figure 8. It is obvious from Figure 8 that all of the Nyquist plots display only a capacitive loop and the plot size augmented with the increase of the inhibitor, demonstrating that the corrosion route is mainly measured by a charge transfer method [42]. Consequently, this behavior is usually revealed when we have the distribution frequency ascribed to the roughness and heterogeneity of the electrode surface.

**Figure 9:**
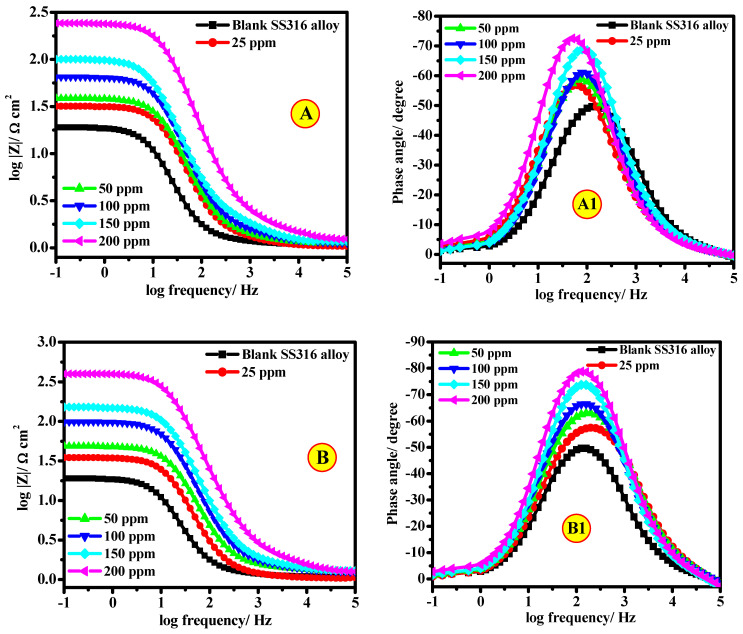
Bode (**A**,**B**) and Bode phase (**A1**,**B1**) diagrams of SS316 alloy in 2.0 M HCl in the absence and the presence of various concentrations of (**A**) CEL and (**B**) NCC, at T = 323 K.

Moreover, the EIS results are also displayed in Bode profiles (Figure 9). The Bode phase angle profiles (Figure 9A1,B1) display a sole peak at middle frequencies, demonstrating the occurrence of a single time constant. Furthermore, the Bode diagram attained in the existence of the prepared CEL and NCC molecules exhibited an individual single-phase maximum, representing one single relaxation route. The experimental tendency to rise in the phase angle value on the addition of the investigated CEL and NCC molecules to the HCl medium designates the development of a defensive layer on the SS316 alloy surface. Therefore, the process of charge transfer can have occurred at the interface of alloy/solution [43]. It is correspondingly detected from the Bode profiles that a linear relationship among log(*f*) vs. log|*Z*| was displayed in the sporadic frequency area, representing that the slope value is close to −1 and the phase angle is less than −90°.

**Figure 10:**
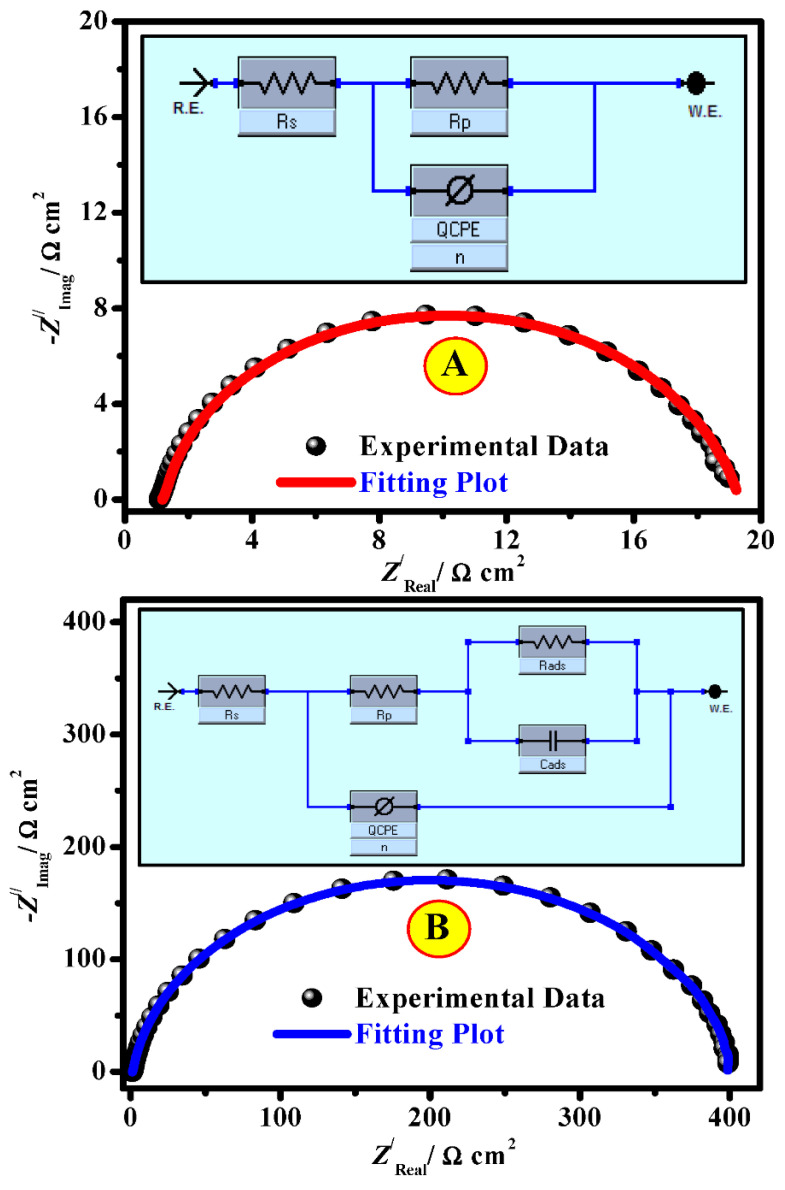
Comparison of empirical Nyquist results (points) and fitted data (line) measured the SS316 alloy in the absence (**A**) and the existence of 200 ppm NCC (**B**). Inset equivalent circuit for the corrosion performance of investigated systems’ (**A**) uninhibited medium and (**B**) inhibited medium.

To characterize the SS316 alloy/medium interface, the model of an electrical equivalent circuit (EEC) is applied to obtain information about the double-layer. Comparison of experiential results of Nyquist plots (points) and fitted findings (line) measured the SS316 alloy in the inhibited medium (A) and inhibitor-containing 200 mg/L CEL (B) and EEC for the corrosion process of studied systems (A) blank system, and (B) inhibited solutions were depicted in Figure 10A,B (inset).

A physical depiction of the EEC comprises the solution ohmic resistance (*R*_s_), constant phase element (*Q*_CPE_), and polarization resistance (*R*_p_). In the case of solution-containing inhibitors, *R*_p_ is in series to the parallel of capacitance due to the inhibitor film adsorption (*C*_ads_) and the resistance because of inhibitor film adsorption (*R*_ads_). Due to the electrode surface inhomogeneity, the double layer modelization was advanced using a CPE rather than the capacitance of double-layer (*C*_dl_) [44]. In this circumstance, CPE accurately accorded the impedance of the metal/electrolyte interface in place of the capacitor. The empirical findings were fitted, and the pretend indices based on the EEC were collected in Table 2. The documented Chi-square (*χ*^2^) values achieved from the EEC fitting were up to 59.7 × 10^−5^ (Table 2). These minor values of *χ*^2^ propose the superiority of fitting the suggested EEC and experiential results.

The organized outcomes (Table 2) presented an upsurge in the *R*_p_ values from 19.47 Ω cm^2^ (for the uninhibited medium) to values of 248.28 and 398.72 Ω cm^2^ with the addition of 200 mg/L of CEL and NCC molecules, respectively, which match the capability of the molecules to hinder charge transfer across interface SS316 alloy/solution, as well as the protection capacity, which accomplished a maximum value of 92.1% for CEL and 95.1% for NCC at the maximum-investigated dose (200 mg/L). Conversely, the *C_dl_* and *Y*_0_ values declined as both molecules’ concentrations augmented, demonstrating adsorption on the SS316 alloy surface. Furthermore, the obtained values of *n* are lower than unity in both the blank and solution-containing inhibitors, which designates that the element of CPE performs as a pseudo capacitor [45]. The inhibitory efficacies of these prepared CEL and NCC molecules are related to the occurrence of -OH groups as active centers attached to the aromatic nucleus of these compounds. The obtained outcomes display CEL and NCC molecule performances as good inhibitors for SS316 alloy corrosion in the investigated corrosive medium.

Table 3 shows the percentage protection efficacy for some designated cellulose applied as inhibitors for steel alloys corrosion compared with our polymers (CEL and NCC). The protection power values, recorded in Table 3 [46,47,48], were achieved using the PDP method. By comparing these findings, we could display that our natural cellulose derivatives, CEL and NCC, are the most efficient inhibitors for the SS316 alloy in 2.0-N hydrochloric acid. Furthermore, natural cellulose derivatives continue to be effective against the SS316 alloy corrosion at high temperatures (93.1% at 50 °C).

#### 3.2.5. Adsorption Isotherm Analysis

Corrosion inhibitors mostly procedure sole or several films on metal surfaces, which assist as a barrier on the electrode interface and therefore protect it from corrosive attack. The formation layer mechanism can be via the inhibitor molecule adsorption on the electrode interface through chemical mode, physical mode, or both mechanisms. The findings from PDP analysis were utilized to appreciate the adsorption mode of CEL and NCC compounds. Numerous models of adsorption isotherms, such as the Temkin, Langmuir, and Freundlich, were evaluated. It was established that the preeminent appropriate adsorption was attained by using the Langmuir model. This model approves monolayer adsorption that is independent and homogeneous, which is given by the following Equation [49]:

(2) $$\frac{C_{\mathrm{inh}}}{\theta }=\frac{1}{K_{\mathrm{ads}}}+(a_{L})C_{\mathrm{inh}}$$

where *C*_inh_ is [CEL and NCC], *θ* represents the part of surface coverage, *K*_ads_ is the constant for equilibrium adsorption route, and *a_L_* is the Langmuir isotherm constant (slope). 

**Figure 11:**
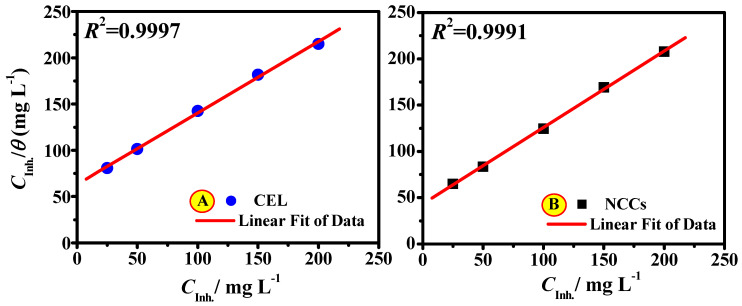
Langmuir adsorption isotherms for adsorption of (**A**) CEL and (**B**) NCC on the SS316 alloy in 2.0 M HCl.

The diagram of *C*_inh_ vs. *C*_inh_/*θ* provides a linear line with the slope as *a_L_* and intercept as 1/*K*_ads_. The correlation coefficient values (*R*^2^): 0.9997 and 0.9991 for CEL or NCC compounds, respectively, are acquired from the straight-form diagrams (Figure 11), and this is a suggestion that the findings followed the Langmuir model. The *K*_ads_ values of the CEL or NCC molecules are 0.0632 and 0.043 L/g, respectively, attained from the intercept of a graph (Figure 11). *K*_ads_ is correlated with $\Delta G_{ads}^{0}$ the Gibbs free energy of adsorption as expressed in the following Equation [50]:

(3) $$\Delta G_{ads}^{0}=-RT\ln(1\times 10^{6}K_{\mathrm{ads}})$$

where: *R* = 8.314 J mol^−1^ K^−1^, *T* is Kelvin temperature and the value 1 × 10^6^ is the water concentration.

The $\Delta G_{ads}^{0}$ values ~ −20 kJ/mol are related to the physical adsorption mechanism. This model is a bonding route among the metal residing atoms on the metal surface and the inhibitor molecules, while values of $\Delta G_{ads}^{0}$ ~ −40 kJ/mol are interrelated with the chemical adsorption mechanism. This mode of adsorption includes the contribution of a pair of electrons from the inhibitor molecule to the empty d-orbitals of the iron, whereas $\Delta G_{ads}^{0}$ values between −20 and −40 kJ/mol propose both adsorption modes [51]. Herein, the values of $\Delta G_{ads}^{0}$ are found to be −44.52 and −45.53 kJ/g for CEL or NCC molecules, respectively, demonstrating the adsorption of NCC or CEL to the metal interface to have occurred via chemical adsorption mode.

#### 3.2.6. Surface Analysis and Characterization 

**Figure 12:**
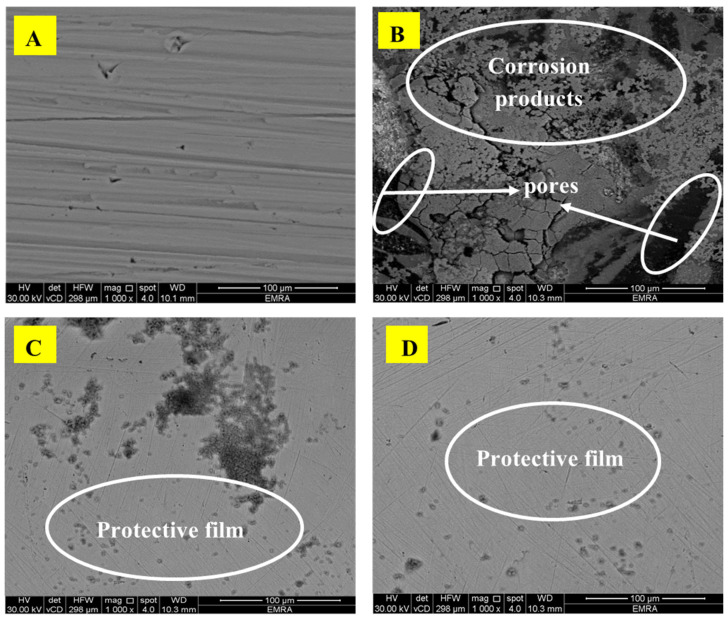
SEM pictures of the SS316 alloy surface; (**A**) before dipping; (**B**) after dipping in 2.0 M HCl solution-free inhibitor; (**C**) after dipping in 2.0 M HCl + 200 mg/L CEL; (**D**) after dipping in 2.0 M HCl + 200 mg/L NCC. Exposure time = 48 h, at 323 K.

To examine the SS316 alloy interface, the specimens were immersed in 2.0 M HCl with and without the CEL and NCC compounds, for 48 h, at 323 K (Figure 12A–D). As it could be observed from (Figure 12A), the SEM picture shows the pristine polished SS316 alloy. The unprotected electrode dipped in the corrosive medium-free inhibitor exhibits surface roughness with cracks and dips randomly distributed over the entire surface (Figure 12B). Figure 12C,D display images of the alloy specimens immersed in the aggressive medium-containing CEL and NCC inhibitors, respectively. These graphs exhibited fewer surface cracks, pits, and roughness compared to the substrate dipped in the blank solution. The SS316 alloy interface immersed in 2.0 M HCl-containing NCC molecules presented a comparatively smoother interface and less pitted morphology than the steel immersed in the inhibited HCl solution-containing CEL molecules. This variance is a consequence of the defensive layer of the NCC inhibitor, which assists more than CEL as a protective film, which prevents the aggressive ions to reach the electrode surface. The outcomes are matched with the other findings performed by weight loss, PDP, and EIS measurements.

### 3.3. Theoretical Approaches

#### 3.3.1. DFT Studies 

**Figure 13:**
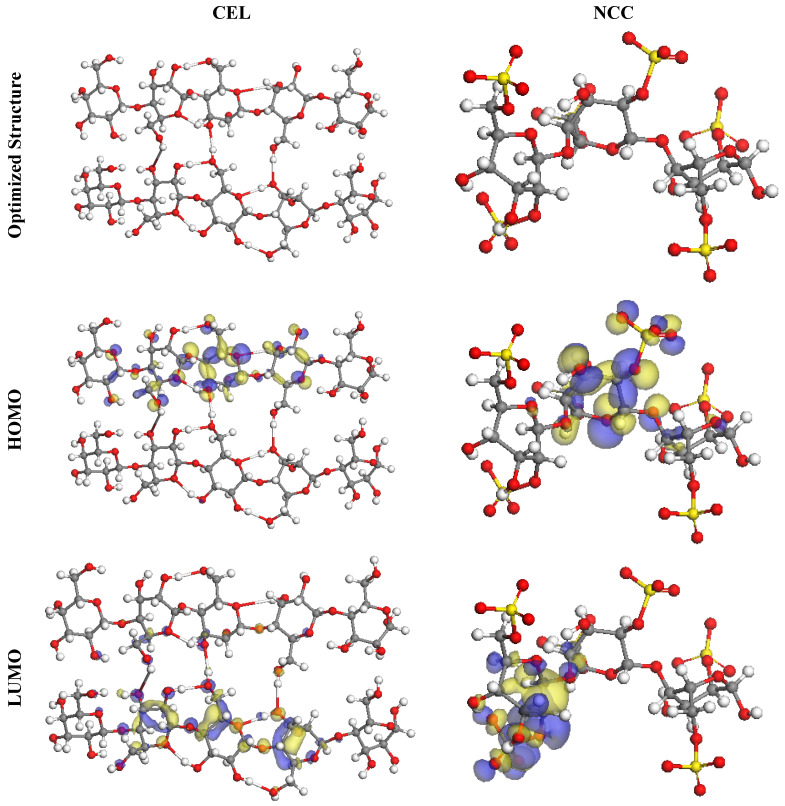
The optimized molecular structures, HOMO and LUMO, of the CEL and NCC using DMol3 module.

DFT studies were executed to inspect the extent of interaction between active centers of CEL and NCC molecules with the SS316 alloy surface. The optimized structures, HOMO and LUMO orbitals for CEL and NCC, are revealed in Figure 13, and the related quantum chemical parameters are listed in Table 4. According to the FMO theory, the donor or acceptor capabilities of an inhibitor molecule at the interface of inhibitor/metal surface are appointed by HOMO and LUMO energies [52]. As follows, the molecule with high *E*_HOMO_ and low *E*_LUMO_ values is considered a highly effective corrosion inhibitor. As described in Table 4, the NCC has the maximum *E*_HOMO_ value of −5.45 eV as compared to CEL (−5.73 eV). As exhibited in Figure 13, for the inhibitor molecules, it is evident that the HOMO level was located on the pyran, hydroxy, and sulfate moieties, implying that the oxygen and sulfur atoms are the desired sites for electrophilic attacks on the iron surface. These explanations endorse the proficiency of inhibitor molecules for adsorption on the SS316 alloy surface and consequently boost the inhibition efficacy, which accords with the experimental results. On the contrary, the *E*_LUMO_ values are 0.10 eV for CEL (Table 4), smaller than NCC (0.33 eV), but this is incompatible with the experimental results, whereas NCC has higher inhibition performance than CEL. Likewise, the energy gap (Δ*E*) is a pivotal indicator to reinforce the corrosion protection proficiency of the inhibitor molecule, which rises as the Δ*E* value is lessened [53]. As evidenced in Table 4, NCC has a smaller Δ*E* value (5.78 eV) than CEL (5.83 eV), which supports a higher trend of NCC to be adsorbed on the SS316 alloy surface. Furthermore, the little values of electronegativity (χ) also offer a large potential reactivity of the inhibitor molecules to afford electrons to the metal surface, so NCC has a lower value of χ (2.56) than CEL (2.82), indicating the high adsorption for NCC [54]. Additionally, the stability and reactivity of the molecule can measure from hardness (η) and softness (σ), i.e., soft molecules have more protecting ability than hard molecules by virtue of the smooth delivery of electrons to the metal surface through adsorption; therefore, they are considered effective corrosion inhibitors [55]. As revealed in Table 4, NCC tends to have lower η values and higher σ values than CEL, and this clearly indicates the great inhibitor ability for a contribution of electrons to the metal surface and high inhibition efficacy.

The Δ*N* values evaluate the propensity of a molecule to provide the metal surface with electrons, and the higher the Δ*N* value, the greater ability the of the inhibitor molecule to contribute the electrons [56]. Pursuant to the evaluation values of Δ*N* are recorded in Table 4, NCC (0.77) has higher ΔN values than CEL (0.72). This suggests that NCC has a superior inclination to afford electrons to the metal surface than CEL. Furthermore, when η > 0, the Δ*E*_back-donation_ will be < 0, the electron transferred to a molecule, pursued by a back-donation from the molecule, and this is energetically preferred [30]. In Table 4, the computed Δ*E*_back-donation_ values for CEL and NCC are negative (−0.73, −0.72), which reveals that back-donation is favored for the CEL and NCC, forming a strong bond with the SS316 alloy surface [57].

Moreover, the dipole moment is a critical indicator that assists in foretelling the path of corrosion inhibition [58]. The raise in the dipole moment affords improvement in the deformation energy and augments the molecule adsorption on the metal surface. Therefore, the increment in the dipole moment gives rise to improve in corrosion inhibition efficacy [59]. As demonstrated in Table 4, NCC has a larger dipole moment value (16.64 debye) than CEL (10.24 debye), which confirms the higher proclivity for NCC to be adsorbed on the SS316 alloy surface and improve the inhibition.

Additionally, the predilection of inhibitor molecules to conserve the SS316 alloy surface in corrosive environments is related to their molecular surface area. The inhibition efficacy increases as the size of the molecular structure increases as the interface area between the inhibitor molecules and the SS316 alloy surface rises. As designated in Table 4, CEL exhibits the highest molecular surface area, but this contradicts with experimental results, whereas the great inhibition proficiency is greater for NCC (343.96 ${Å}^{2}$) than CEL (1222.34 ${Å}^{2}$), and this may be due to the high steric hindrance for the CEL molecule, which decreases its adsorption on the SS316 alloy surface.

**Figure 14:**
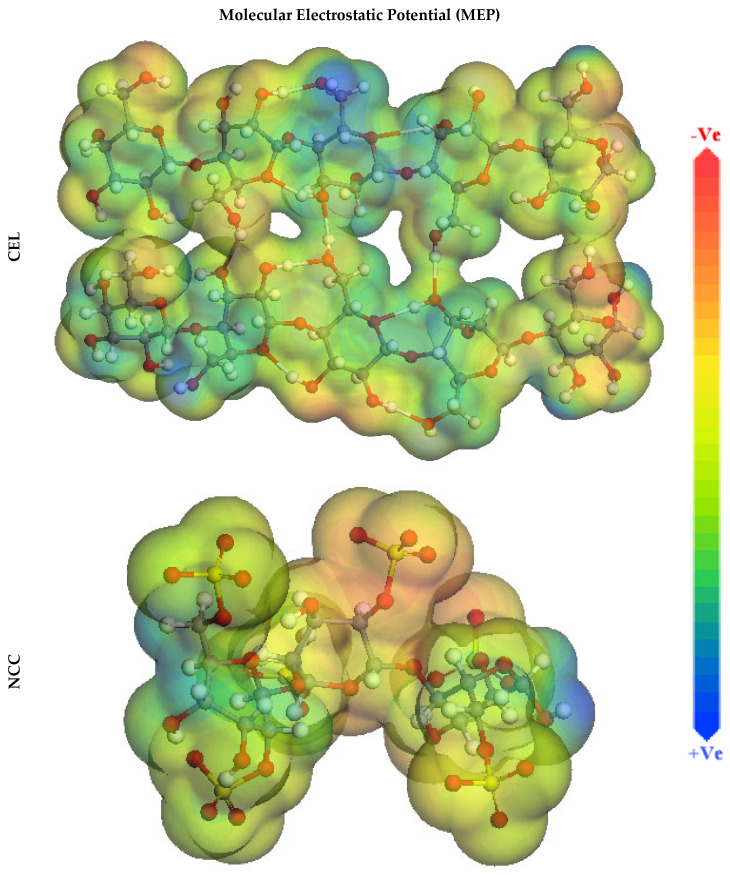
Graphical presentation of the MEP of the CEL and NCC using DMol3 module.

In addition, molecular electrostatic potential mapping (MEP) can scout the active centers of inhibitor molecules and be reckoned to exploit the Dmol^3^ module. The MEP mapping is a 3D visual descriptor meant to identify the net electrostatic influence founded upon a molecule by the overall charge dispensation [60]. According to the MEP maps exhibited in Figure 14, the red colors portray the maximum electron density region wherever the MEP is maximum negative (nucleophilic reaction). In contrast, the blue colors describe the greatest positive area (electrophilic reaction) [61]. An optical inspection of Figure 14 endorses that the highest negative areas are mainly over hydroxy and sulfate moieties, whereas the lesser electron density above the pyran moieties in inhibitor molecules. Conversely, MEP disclosed the maximum probable positive area over hydrogen atoms in inhibitor molecules. These sites with excessive electron density (i.e., red regions) in inhibitor molecules could be the most appropriate for interactions with the SS316 alloy surface, forming a highly adsorbed shielding layer.

#### 3.3.2. MC Simulations

**Figure 15:**
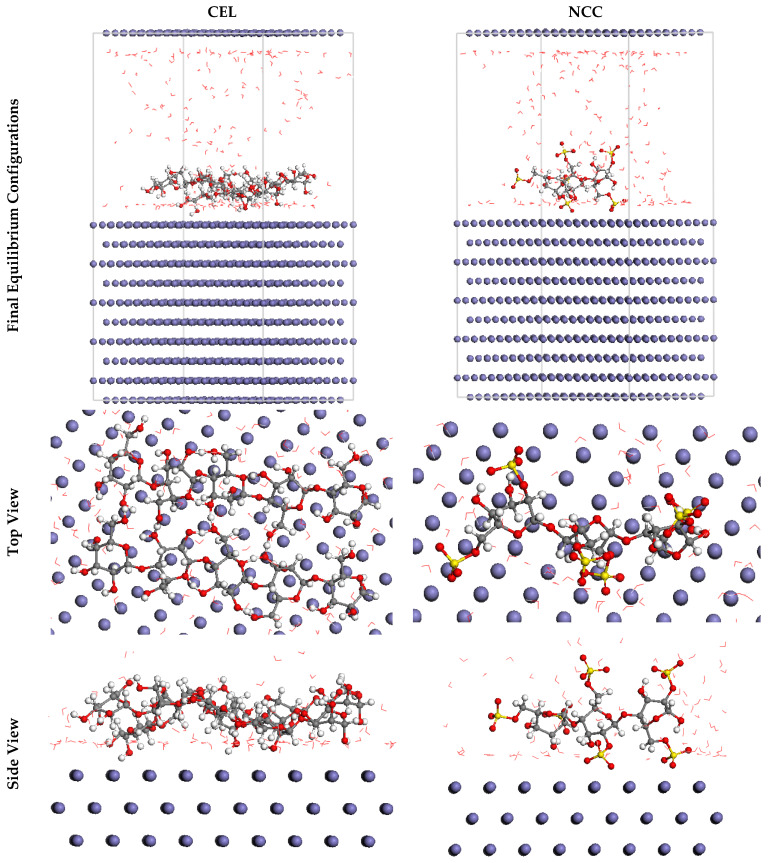
The most suitable configuration for adsorption of the **CEL** and **NCC** on Fe (1 1 0) substrate obtained by adsorption locator module.

MC simulations were devoted to proposing a manifest concept for the mechanism of adsorption as well as recognizing the interactions of the inhibitor molecules with the SS316 alloy surface. Subsequently, Figure 15 exposes the greatest proper adsorption configurations for the inhibitor molecules on the SS316 alloy surface achieved by the adsorption locator module, which presents in nearly plane arrangements, implying an i improvement in the adsorption and highest surface coverage [62]. Furthermore, the attained outcomes for the adsorption energies reckoned from the MC simulations were exhibited in Table 5. As divulged in Table 5, NCC (−2831.49 kcal mol^−1^) has a higher negative value of the adsorption energy as compared to the CEL (−2133.54 kcal mol^−1^), which postulates forceful adsorption of NCC on the SS316 alloy surface, creating a firmly adsorbed layer, and resists the corrosion of SS316 alloy; these results are compatible with the experimental outcomes [63]. Additionally, Table 5 shows that the adsorption energies values of NCC for the pre-geometry optimization step, i.e., unrelaxed (−2962.22 kcal mol^−1^), are more negative than CEL (−2194.93 kcal mol^−1^) and for the post-geometry optimization step, i.e., relaxed (130.73 kcal mol^−1^, correspondingly), are higher than CEL (61.39 kcal mol^−1^), asserting a higher inhibition proficiency for NCC than CEL.

The d*E*_ads_/d*N*_i_ values elucidate the metal-adsorbates configuration energy if adsorbed inhibitor or water molecules have been omitted [64]. The d*E*_ads_/d*N*_i_ values for NCC (−1297.24 kcal mol^−1^) are greater than CEL (−303.04 kcal mol^−1^), as exhibited in Table 5, which declares an excellent adsorption of NCC than CEL. Moreover, the d*E*_ads_/d*N*_i_ values for water nearby −15.19 kcal mol^−1^ are low in comparison with CEL and NCC values, revealing the more burly adsorption of inhibitor molecules than water molecules, which supports the exchange of water molecules by inhibitor molecules. Therefore, the CEL and NCC are resolutely adsorbed on the SS316 alloy surface and form a powerful adsorbed protective layer that offers corrosion resistance for the SS316 alloy surface in corrosion media; this was affirmed by empirical and computational studies jointly.

## 4. Conclusions

Herein, the comparative computational and empirical inhibition studies of macrocrystalline and nanocrystalline cellulose on the SS316 alloy corrosion during the acid pickling process were investigated. Nanocrystalline cellulose was prepared by acid treatment of macrocrystalline cellulose. The prepared nanocrystalline cellulose was characterized using FTIR, FE-SEM, BET surface area, TEM, and Raman spectroscopy. The morphologies of nanocrystals were shown, supporting proof with TEM examination of NCC of nano-scale range. The NCC had a normal size of rod-like nanocrystals, 100–200 nm in length and 10–20 nm in diameter. NCC and CEL polymers act as efficient inhibitors for SS316 alloy corrosion, and their efficacies upsurge with augmented concentration. Maximum protection capacities of 93.1% and 96.3% were observed for CEL and NCC, respectively. PDP study suggested that CEL and NCC acted as mixed-inhibitor types. SEM analyses exhibited that the polymers adsorb on the metal interface and impede the SS316 alloy corrosion in HCl solution, and their adsorption on the metal substrate followed the Langmuir isotherm model. DFT calculation displayed that CEL and NCC interact with the metal interface by donor–acceptor attractions. Moreover, the MC simulations study exhibited that CEL and NCC impulsively adsorb via their electron-rich sites.

## Figures and Tables

**Table 1:** PDP parameters obtained for SS316 alloy in 2.0 M HCl solution in the lack (blank) and existence of different concentrations of CEL and NCC at 50 °C.

Inhibitors Code	*C*_inh_/ppm	*E*_cor_/V vs. (Ag/AgCl)	*j*_cor_/µA cm^−2^ ± SD	*β*_a_/mV dec^−1^ ± SD	−*β*_c_/mV dec^−1^ ± SD	*θ*	*ζ*_PDP_/%
Blank	0.0	−0.350	1054.5 ± 65	92.4 ± 6.6	172.3 ± 13.1	-	-
**CEL**	25	−0.346	728.6 ± 24	93.2 ± 4.1	169.5 ± 15.3	0.309	30.9
	50	−0.345	534.6 ± 21	89.3 ± 5.2	171.3 ± 14.3	0.493	49.3
	100	−0.343	314.2 ± 15	90.5 ± 6.3	166.4 ± 12.8	0.702	70.2
	150	−0.354	183.4 ± 9	92.8 ± 3.9	162.5 ± 11.4	0.826	82.6
	200	−0.334	72.76 ± 4	95.9 ± 4.8	177.6 ± 10.8	0.931	93.1
**NCC**	25	−0.353	648.5 ± 23	96.7 ± 6.5	176.6 ± 14.7	0.385	38.5
	50	−0.332	422.8 ± 13	98.4 ± 6.4	177.9 ± 12.7	0.599	59.9
	100	−0.344	208.7 ± 11	90.5 ± 5.4	172.8 ± 15.3	0.802	80.2
	150	−0.343	126.4 ± 7	99.7 ± 7.1	173.7 ± 11.8	0.887	88.7
	200	−0.346	39. 1 ± 2	91.8 ± 7.2	177.6 ± 16.2	0.963	96.3

**Table 2:** EIS parameters obtained for SS316 alloy in 2.0 M HCl solution in the lack (blank) and existence of different concentrations of CEL and NCC at 50 °C.

Inhibitor Codes	*C*_inh_/ppm	*R*_e_/Ω cm^2^	*R*_P_/Ω cm^2^	*C_dl_*/µ F cm^−2^	*Q*_CPE_		*χ*^2^ × 10^−4^	*θ*	*ζ*_E_/%
					*Y*_0_/μΩ^−1^ s^n^ cm^−2^	*n*			
Blank	0.0	0.96	19.47	760.25	62.38	0.776	4.86	-	-
**CEL**	25	1.14	32.25	232.49	18.29	0.851	4.97	0.396	39.6
	50	1.15	39.38	130.15	10.21	0.881	5.03	0.505	50.5
	100	1.25	66.61	85.87	6.59	0.825	5.09	0.707	70.7
	150	1.26	103.81	75.55	5.53	0.871	5.29	0.812	81.2
	200	1.28	248.28	52.24	4.94	0.831	5.15	0.921	92.1
**NCC**	25	1.18	34.95	227.14	17.69	0.819	4.89	0.443	344.0
	50	1.15	48.64	118.71	9.89	0.866	5.19	0.599	59.9
	100	1.28	98.66	63.18	4.932	0.873	5.33	0.802	80.2
	150	1.28	152.35	49.93	3.24	0.898	5.38	0.872	87.2
	200	1.29	398.72	37.97	2.89	0.888	5.97	0.951	95.1

**Table 3:** Comparison of the corrosion protection capacity of the as-fabricated CNC, Cu-M@CNC, and Ni-M@CNC nanocomposites on steel alloys with some previous studies.

Inhibitor Name	Corrosive Medium	Optimum Concentration	Max. Protection Capacity/%	Refs.
Chitosan	CO_2_-saturated 3.5% NaCl solution	100 ppm	45	**Ref.** [46]
Commercial inhibitor	CO_2_-saturated 3.5% NaCl solution	100 ppm	88	**Ref.** [46]
CMC/AgNPs Composite	15% H_2_SO_4_	1000 ppm	89.9	**Ref.** [47]
Aminated hydroxyl ethyl cellulose	1.0 M HCl	900 ppm	91	**Ref.** [48]
CEL	2.0 M HCl	200 ppm	93.1	**Current work**
NCC	2.0 M HCl	200 ppm	96.3	**Current work**

**Table 4:** The calculated quantum chemical parameters for the **CEL** and **NCC**.

Inhibitor	CEL	NCC
*E*_HOMO_, eV	−5.73	−5.45
*E*_LUMO_, eV	0.10	0.33
∆*E*, eV	5.83	5.78
*I*	5.73	5.45
*A*	−0.10	−0.33
*χ*	2.82	2.56
*η*	2.92	2.89
*σ*	0.34	0.35
Δ*N*	0.72	0.77
∆*E*_back-donation_, eV	−0.73	−0.72
Dipole moment value, debye	10.24	16.64
Molecular surface area, Å^2^	1222.34	343.96

**Table 5:** Data and descriptors calculated by the Monte Carlo simulation (MC) for adsorption of the **CEL** and **NCC** on iron (1 1 0).

Structures	Adsorption Energy/ Kcal mol^−1^	Rigid Adsorption Energy/ kcal mol^−1^	Deformation Energy/ kcal mol^−1^	d*E*_ads_/d*N*_i_: Inhibitor kcal mol^−1^	d*E*_ads_/d*N*_i_: Water kcal mol^−1^
Fe (110)	−2133.54	−2194.93	61.39	−303.04	−15.82
**CEL**					
water					
Fe (110)	−2831.49	−2962.22	130.73	−1297.24	−14.56
**NCC**					
water					

## Data Availability

The raw/processed data generated in this work are available upon request from the corresponding author.
